# A Profile of Influenza Vaccine Coverage for 2019-2020: Database Study of the English Primary Care Sentinel Cohort

**DOI:** 10.2196/39297

**Published:** 2024-05-24

**Authors:** Uy Hoang, Gayathri Delanerolle, Xuejuan Fan, Carole Aspden, Rachel Byford, Mansoor Ashraf, Mendel Haag, William Elson, Meredith Leston, Sneha Anand, Filipa Ferreira, Mark Joy, Richard Hobbs, Simon de Lusignan

**Affiliations:** 1 Nuffield Department of Primary Care Health Sciences University of Oxford Oxford United Kingdom; 2 CSL Seqirus Maidenhead United Kingdom; 3 CSL Seqirus Amsterdam Netherlands

**Keywords:** medical records systems, computerize, influenza, influenza vaccines, sentinel surveillance, vocabulary controlled, general practitioners, general practice, primary health care, vaccine, public health, surveillance, uptake

## Abstract

**Background:**

Innovation in seasonal influenza vaccine development has resulted in a wider range of formulations becoming available. Understanding vaccine coverage across populations including the timing of administration is important when evaluating vaccine benefits and risks.

**Objective:**

This study aims to report the representativeness, uptake of influenza vaccines, different formulations of influenza vaccines, and timing of administration within the English Primary Care Sentinel Cohort (PCSC).

**Methods:**

We used the PCSC of the Oxford-Royal College of General Practitioners Research and Surveillance Centre. We included patients of all ages registered with PCSC member general practices, reporting influenza vaccine coverage between September 1, 2019, and January 29, 2020. We identified influenza vaccination recipients and characterized them by age, clinical risk groups, and vaccine type. We reported the date of influenza vaccination within the PCSC by International Standard Organization (ISO) week. The representativeness of the PCSC population was compared with population data provided by the Office for National Statistics. PCSC influenza vaccine coverage was compared with published UK Health Security Agency’s national data. We used paired *t* tests to compare populations, reported with 95% CI.

**Results:**

The PCSC comprised 7,010,627 people from 693 general practices. The study population included a greater proportion of people aged 18-49 years (2,982,390/7,010,627, 42.5%; 95% CI 42.5%-42.6%) compared with the Office for National Statistics 2019 midyear population estimates (23,219,730/56,286,961, 41.3%; 95% CI 4.12%-41.3%; *P*<.001). People who are more deprived were underrepresented and those in the least deprived quintile were overrepresented. Within the study population, 24.7% (1,731,062/7,010,627; 95% CI 24.7%-24.7%) of people of all ages received an influenza vaccine compared with 24.2% (14,468,665/59,764,928; 95% CI 24.2%-24.2%; *P*<.001) in national data. The highest coverage was in people aged ≥65 years (913,695/1,264,700, 72.3%; 95% CI 72.2%-72.3%). The proportion of people in risk groups who received an influenza vaccine was also higher; for example, 69.8% (284,280/407,228; 95% CI 69.7%-70%) of people with diabetes in the PCSC received an influenza vaccine compared with 61.2% (983,727/1,607,996; 95% CI 61.1%-61.3%; *P*<.001) in national data. In the PCSC, vaccine type and brand information were available for 71.8% (358,365/498,923; 95% CI 71.7%-72%) of people aged 16-64 years and 81.9% (748,312/913,695; 95% CI 81.8%-82%) of people aged ≥65 years, compared with 23.6% (696,880/2,900,000) and 17.8% (1,385,888/7,700,000), respectively, of the same age groups in national data. Vaccination commenced during ISO week 35, continued until ISO week 3, and peaked during ISO week 41. The in-week peak in vaccination administration was on Saturdays.

**Conclusions:**

The PCSC’s sociodemographic profile was similar to the national population and captured more data about risk groups, vaccine brands, and batches. This may reflect higher data quality. Its capabilities included reporting precise dates of administration. The PCSC is suitable for undertaking studies of influenza vaccine coverage.

## Introduction

The range of influenza vaccines available for clinical use has grown; there are now live attenuated influenza vaccines and recombinant vaccines in addition to the long-established inactivated influenza vaccine [1,2]. Live attenuated influenza vaccines have also been widely introduced to children via intranasal administration [3,4]. There have also been many new formulations of inactivated influenza vaccine (IIV). IIV are now often quadrivalent (active against 2 influenza B lineages, as well as 2 influenza A subtypes) rather than trivalent (which has a single B lineage), and their effectiveness may be increased by the addition of an adjuvant or higher antigen dosages [4]. Cell-based manufacture of IIV has been introduced in addition to traditional egg-based methods. Recombinant influenza vaccines are also being introduced, although these formulations are currently less widely used [5,6]. The available evidence indicates additional benefits from the use of newer and enhanced influenza vaccine formulations [7].

The Joint Committee on Vaccination and Immunization (JCVI) provides UK-wide advice on vaccination policy, including for seasonal influenza vaccine. The JCVI has progressively updated its recommendations as new vaccines have been introduced. These include the use of adjuvanted trivalent inactivated influenza vaccine (aIIV3) and high-dose trivalent inactivated influenza vaccine (HD-IIV3) for use in the 2018-2019 season and the use of cell-based quadrivalent inactivated influenza vaccine (IIV4c) vaccine for the 2019-2020 influenza season [8]. IIV4c was approved for adults and children from 9 years of age in at-risk groups and was available for use starting in the 2019-2020 season in the United Kingdom [9]. For the 2021-2022 season, a non-egg–based quadrivalent vaccine based on recombinant technology was added to the recommendations put forward by the JCVI for adults aged between 18 and 65 years [7]. For adults aged 65 years or older, 2 vaccine formulations were recommended by the JCVI in the 2021-2022 season, namely aIIV4, which replaced the trivalent formulation; aIIV3; and high-dose quadrivalent inactivated influenza vaccine (HD-IIV4) [7].

Additionally, the JCVI advised that the relative effectiveness of different enhanced influenza vaccine formulations should be assessed. Comparative influenza vaccine effectiveness data are required, preferably from the same country over multiple seasons and with laboratory-confirmed influenza end points [7,10].

In the context of an ever-increasing range of influenza vaccine formulations, we report influenza vaccine coverage in the English Primary Care Sentinel Cohort (PCSC) in the 2019-2020 winter season. We describe the representativeness of the PCSC compared with the national population, the uptake of influenza vaccination within this cohort (differentiated by formulation and also compared with national data), and the timing of vaccine administration across age bands.

## Methods

### Study Design and Setting

In this study, we compare the PCSC with national data for population representativeness and vaccine uptake by formulation and provide a descriptive report by week of vaccine uptake. These elements were included because vaccine studies can either be analyzed at the national population level or run in representative samples. It is important to know if there is sufficient representation of the different influenza vaccine formulations and to understand the timings of vaccine administration for studies of adverse events of interest or those of vaccine effectiveness compared with known dates of cocirculating strains of influenza [11].

This study was conducted during the 2019-2020 influenza season. The 2020-2021 season was not used as it was considered by authors as atypical due to the COVID-19 pandemic and associated changes in vaccination schedules (inclusion of persons aged 50 to 64 years and an absence of circulating influenza as a result of stay-at-home mandates) [12]. This study was conducted using the PCSC of the Oxford Royal College of General Practitioners (RCGP) Research and Surveillance Centre (RSC). At the time of this study, the RSC was, and remains, one of the largest and longest-established primary care sentinel networks in Europe [13,14].

**Figure 1:**
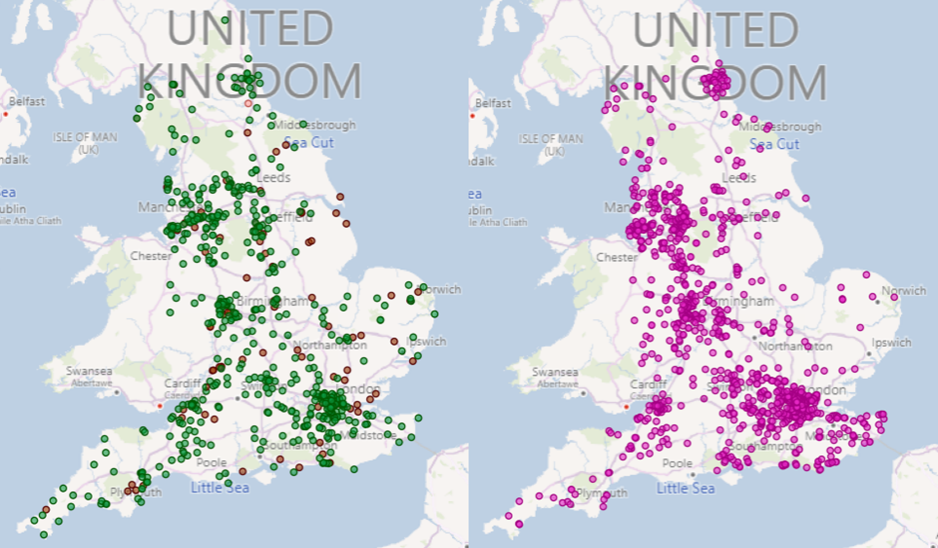
The general practice member of the Oxford-Royal College of General Practitioners Research and Surveillance Centre. The map on the left shows the Primary Care Sentinel Cohort with newly recruited practices to improve national coverage in brown. The right-hand map shows the Syndromic Surveillance General Practices (SSGPs).

At the time of this study, the RSC had been recording respiratory conditions, including influenza and notifiable diseases, and had provided data to support clinical and public health research (most notably on vaccine effectiveness) for over 50 years [3,13-16]. Data from the RSC were, and still are, hosted within the Oxford RCGP Clinical Informatics Digital Hub, a trusted research environment [17]. The RSC as a whole comprises over 1800 general practices in England, and the total current registered list includes over 19 million people—approximately 32% of the national population (Figure 1).

The RSC adapted and grew over the course of the COVID-19 pandemic when it was divided into 2 divisions [18]. First, the PCSC is the long-standing sentinel group of general practices (n=693) that conduct virology sampling (n=240) and serosurveillance (n=273) [19,20]. This group of practices has a long history of receiving feedback about data quality, historically at an annual visit and, in recent years, through dashboards, webinars, and web-based visits [21,22]. They were recruited to be nationally representative [13]. The second group, the Syndromic Surveillance General Practices (SSGPs), was recruited to support the UK Health Security Agency’s (UKHSA; formerly Public Health England) syndromic surveillance [23]. These practices were recruited to complement other data sources and were not nationally representative.

### Representativeness of the PCSC, Including Risk Groups

The study population included participants of all ages in England who were registered with a general practitioner (GP) in the PCSC between September 1, 2019, and January 29, 2020. We describe the demographic characteristics of the study population in terms of the following:

- Age, including the following subgroups: ≤1 year, ≥2 to ≤3 years, ≥4 to ≤17 years, ≥18 to ≤49 years, ≥50 to ≤64 years, and ≥65 years.
- Sex: male, female, and missing.
- Race: Asian, Black, White, mixed, and missing or other, using an ontology to maximize identification [24].
- Socioeconomic status using the quintile of the Index of Multiple Deprivation (IMD) as a proxy (a nationally available measure assigned based on postcode). This provides an overall relative measure of deprivation for each Lower Layer Super Output Area (LSOA). An LSOA is a small area with an average population of 1500 people. The overall deprivation scores are ranked for all LSOAs within a country and can be divided into 5 groups (quintiles), where quintile 1 represents the most deprived LSOAs and quintile 5 represents the least deprived LSOAs. The IMD is a score based on the area as a whole and not everyone within an LSOA necessarily experiences the same level or type of deprivation [25,26].
- Geographic location is defined according to National Health Service (NHS) region [27].
- Risk factors for influenza as defined by the Chief Medical Officer, include chronic pulmonary disease, asthma, coronary health disease, diabetes, liver disease, chronic kidney disease, neurological conditions, immunosuppression, asplenia, learning difficulties, severe mental illness, and obesity [28].

To evaluate the representativeness of the PCSC in terms of the demographic characteristics above, we undertook a quantitative comparison of the age, sex, and IMD profile of the PCSC against published data for 2019-2020 from the Office for National Statistics (ONS) for England [29]. We compared the ethnic profile of the PCSC against ONS ethnic population estimates extrapolated from the 2011 census [30]. We also compared the broad geographic distribution of the PCSC population in terms of NHS regions with the data from the ONS on the geographical distribution of the population by government office regions [27,31]. Last, we compared the distribution of people with risk factors for influenza defined by the Chief Medical Officer in the PCSC with data from the Quality and Outcomes Framework 2019-2020 primary care disease prevalence figures published by NHS Digital [28,32]. We made 1 near-match using the Quality and Outcomes Framework prevalence of chronic obstructive pulmonary disease to match with chronic lung disease in our data. The latter has a broader definition including conditions such as bronchiectasis.

We took extensive steps to avoid denominator inflation within our data processing. Denominator inflation occurs when patients fail to be deregistered. The existence of a unique national identifier (NHS number) helps address this problem [33], as does the national demographic service, which facilitates the linkage of primary care and secondary care data as well as death certificate data. These include removing cases where there is a completely empty medical record and, based on pseudonymized NHS numbers, only keeping the most recent record where duplicates are identified.

### Influenza Vaccine Coverage

The exposure of interest was influenza vaccination. Influenza vaccination was either recorded as a clinical term for vaccine administration or as a prescription. A large number of doses of influenza vaccines are administered by pharmacies and other community health care workers, although the majority of these vaccines are recorded in the primary care computerized medical record (CMR). Where vaccination was recorded as a clinical term in the CMR, it may not have been specific to the formulation, brand, or batch; when vaccination was prescribed, it was brand and batch specific. Prescriptions were only issued for adult and high-risk children who were vaccinated in their general practice [34]. A list of Systematized Nomenclature of Medicine clinical terms used to identify influenza vaccinations from the CMR of patients in the PCSC is listed in Table S1 in Multimedia Appendix 1 [27,35].

Vaccine coverage in the study population was described by providing the count and proportion (expressed as a percentage) of the PCSC vaccinated against influenza overall and by the following:

- Number and percentage of vaccinated individuals.
- Number and percentage of vaccinated individuals by type of vaccine.
- Timing of vaccination by week and day of the week.
- Number and percentage of vaccinated individuals by geographic location.
- Risk factors for influenza as defined by the Chief Medical Officer [28].

We could not precisely match the geographic locations. Our nearest match was to add East and West Midlands government regions to be equivalent to our Midlands region; we also combined Yorkshire and the Humber and the North East to be equivalent to our North East and Yorkshire regions.

To evaluate the representativeness of influenza vaccine coverage in the PCSC, a quantitative comparison was made to the influenza vaccine coverage for the winter season—September 1, 2019, to January 29, 2020—reported by the UKHSA [35]. We compared the influenza coverage in the PCSC according to the following inclusive age groups reported in national data by the UKHSA: 6 months to 1 year, 2-4 years, 5-15 years, 16-64 years, and ≥65 years. For influenza coverage by type of vaccine, coverage in the PCSC was compared with national data from the UKHSA in the following age groups only: 16-64 years and ≥65 years. We again made some near-matches for at-risk groups: coronary heart disease is compared with chronic heart disease, liver disease with chronic liver disease, neurological disease with chronic neurological disease, and obesity with morbid obesity.

### Influenza Vaccination Timing

In order to evaluate the timing of influenza vaccinations given in the PCSC, we calculated the vaccine coverage overall in the study population by week and day of the week and recorded this information in line with its relevant International Standard Organization (ISO) week number.

### Statistical Methods

For categorical measures, we calculated the frequency and percentage of total study participants observed in each category. For continuous and count variables, we presented the mean, SD, median, and range.

For population proportions, we calculated 95% CI using the normal approximation to the binomial calculation [36]. Paired 2-tailed *t* tests were used to compare populations [37].

Statistical analyses were undertaken using R (version 3.5.1; R Core Team) [38].

### Ethical Considerations

This study was classified as a service evaluation (measuring what standard of care this service achieved) by the Medical Research Council/Health Research Authority decision tool [39], so it did not require formal ethical approval. It was reviewed by the RCGP Approval Committee on January 8, 2021.

## Results

### PCSC Population and Comparison With English National Population

A total of 7,010,627 people (male: n=3,488,789, 49.8%; female: n=3,521,838, 50.2%) were registered with a primary care practice from the PCSC that submitted data for this study (n=693) between September 1, 2019, and January 29, 2020. The study population had a greater proportion of people aged 18-49 years (2,982,390/7,010,627, 42.5%; 95% CI 42.5%-42.6%) compared with the 2019 midyear population estimates from ONS (23,219,730/56,286,961, 41.3%; 95% CI 4.12%-41.3%; *P*<.001; Table 1) [29]. Populations in the most deprived quintile, namely those in IMD quintile 1, were underrepresented in this cohort, and those in the least deprived quintile, namely those in IMD quintile 5, were overrepresented.

**Table 1:** Demographic profile of the Primary Care Sentinel Cohort compared with the English national population.

Characteristics			Study population, 2019-2020 (n=7,010,627), n (%)		2019 ONS^a^ midyear population estimates for England [29] (n=56,286,961), n (%)
Overall population			7,010,627 (100)		56,286,961 (100)
**Age range (years, inclusive)**					
	0-1	158,484 (2.3)		1,262,914 (2.2)	
	2-3	157,857 (2.3)		1,351,731 (2.4)	
	4-17	1,126,575 (16.1)		9,408,923 (16.7)	
	18-49	2,982,390 (42.5)		23,219,730 (41.3)	
	50-64	1,320,621 (18.8)		10,689,947 (19)	
	≥65	1,264,700 (18)		10,353,716 (18.4)	
**Sex**					
	Male	3,488,789 (49.8)		27,827,831 (49.4)	
	Female	3,521,838 (50.2)		28,459,130 (50.6)	
**Race^b^**					
	Asian	496,330 (7.1)		4,143,403 (7.8)	
	Black	210,501 (3)		1,846,614 (3.5)	
	White	4,674,668 (66.7)		45,281,142 (85.4)	
	Mixed	114,846 (1.6)		1,192,879 (2.3)	
	Missing or other	1,514,282 (21.6)		548,418 (1)	
**IMD^c^ quintile**					
	1 (most deprived)	1,245,558 (17.8)		11,267,059 (20)	
	2	1,353,953 (19.3)		11,576,973 (20.6)	
	3	1,409,593 (20.1)		11,424,153 (20.3)	
	4	1,451,445 (20.7)		11,117,694 (20.3)	
	5 (least deprived)	1,548,132 (22.1)		10,901,082 (19.4)	
**Region**					
	London	1,025,002 (14.6)		8,961,989 (15.9)	
	East of England	12,122 (7.3)		6,236,072 (11.1)	
	Midlands	1,000,656 (14.3)		10,769,965 (19.1)	
	North East and Yorkshire	823,102 (11.7)		8,172,908 (14.5)	
	North West	1,077,808 (15.4)		7,341,196 (13)	
	South East	1,339,701 (19.1)		9,180,135 (16.3)	
	South West	1,232,236 (17.6)		5,624,696 (10)	
**Risk factor^d^**					
	Coronary heart disease	647,526 (9.2)		1,891,019 (3.1)^e^	
	Asplenia	36,199 (0.5)		N/A^f^	
	Asthma	488,596 (7)		3,916,150 (6.5)^e^	
	COPD^g^	205,107 (2.9)		1,170,786 (1.9)^e^	
	Chronic kidney disease	267,091 (3.8)		1,949,865 (4.1)^h^	
	Liver disease	107,984 (1.5)		N/A	
	Diabetes	407,228 (5.8)		3,455,176 (7.1)^h^	
	Immunosuppression	136,583 (1.9)		N/A	
	Neurological disease	366,963 (5.2)		N/A	
	Severe mental illness	66,319 (0.9)		562,831 (0.9)^e^	
	Learning difficulties	35,327 (0.5)		308,237 (0.5)^e^	
	Obesity	162,397 (2.3)		5,061,690 (10.5)^h^	

^a^ONS: Office for National Statistics.

^b^Race is recorded in Research and Surveillance Centre data as Asian (9.0%), Black (3.8%), White (85%), and mixed (2.1%).

^c^IMD: Index of Multiple Deprivation.

^d^National prevalence figures for risk factors are based on Quality and Outcomes Framework 2019-2020 prevalence [32].

^e^n=60,407,685.

^f^N/A: not applicable.

^g^COPD: chronic obstructive pulmonary disease.

^h^n=48,146,685.

### Influenza Vaccine Coverage in the PCSC Compared With National Data

A total of 1,731,062 (24.7%; 95% CI 24.7%-24.7%) out of 7,010,627 people within the PCSC received an influenza vaccine (male: 790,660/1,731,062, 22.7%; female: 940,402/1,731,062. 26.7%). We subdivided vaccine coverage in the PCSC population by sociodemographic characteristics (Table 2 and Table S2 in Multimedia Appendix 1) and calculated the proportion of the PCSC population who were vaccinated in each subpopulation stratum. For example, 72.3% (913,695/1,264,700; 95% CI 72.2%-72.3%) of people aged 65 years or older in the PCSC were vaccinated, whereas the national data showed that 72.4% (7,621,505/10,523,854) of individuals aged 65 years or older received seasonal influenza vaccine [35], with a coverage of 24.2% (14,468,665/59,764,928; 95% CI 24.2%-24.2%; *P*<.001). National coverage data only provided information on limited age bands, making direct comparisons between cohorts challenging (see Table S2 in Multimedia Appendix 1). Coverage of risk groups was slightly higher in our study than in comparable national data; for example, 69.8% (284,280/407,228; 95% CI 69.7%-70%) of people with diabetes in our study received an influenza vaccine whereas 61.2% (983,727/1,607,996; 95% CI 61.1%-61.3%; *P*<.001) of people with diabetes received an influenza vaccine according to national data (Table 3).

**Table 2:** Influenza vaccine coverage in strata of age, sex, race, Index of Multiple Deprivation, and region in the Primary Care Sentinel Cohort.

Subgroup		Vaccinated, n/N (%)	Vaccinated (%), 95% CI
**Age (years)**			
	0-1	1216/158,484 (0.8)	0.7-0.8
	2-3	63,532/157,857 (40.3)	40-40.5
	4-17	259,902/1,126,575 (23.1)	23-23.2
	18-49	216,040/2,982,390 (7.2)	7.2-7.3
	50-64	276,677/1,320,621 (21)	20.9-21
	≥65	913,695/1,264,700 (72.3)	72.2-72.3
	All ages	1,731,062/7,010,627 (24.7)	24.7-24.7
**Sex**			
	Male	790,660/3,488,789 (22.7)	22.6-22.7
	Female	940,402/3,521,838 (26.7)	26.7-26.8
**Race**			
	Asian	82,293/496,330 (16.6)	16.5-16.7
	Black	29,884/210,501 (14.2)	14.1-14.4
	White	1,325,892/4,674,668 (28.4)	28.3-28.4
	Mixed	16,797/114,846 (14.6)	14.4-14.8
	Missing or other	276,196/1,514,282 (18.2)	18.2-18.3
**IMD^a^ quintile**			
	1 (most deprived)	246,536/1,245,558 (19.8)	19.7-19.9
	2	297,114/1,353,953 (21.9)	21.9-22
	3	351,594/1,409,593 (24.9)	24.9-25
	4	390,403/1,451,445 (26.9)	26.8-27
	5 (least deprived)	444,876/1,548,132 (28.7)	28.7-28.8
**NHS^b^ region**			
	London	148,930/1,025,002 (14.5)	14.5-14.6
	East of England	131,758/12,122 (25.7)	25.6-25.9
	Midlands	256,769/1,000,656 (25.7)	25.6-25.8
	North East and Yorkshire	226,792/823,102 (27.6)	27.5-27.7
	North West	277,648/1,077,808 (25.8)	25.7-25.8
	South East	337,225/1,339,701 (25.2)	25.1-25.3
	South West	351,940/1,232,236 (28.6)	28.5-28.6

^a^IMD: Index of Multiple Deprivation.

^b^NHS: National Health Service.

**Table 3:** Proportion of risk groups who are vaccinated in the Primary Care Sentinel Cohort (PCSC) compared with national data.

Risk group	PCSC, 2019-2020, n/N (%)	National data [35], n (%)	*P* value^a^
Coronary heart disease vs chronic heart disease	419,417/647,526 (64.8)	432,939/1,042,670 (41.1)	N/A^b^
Asplenia	19,895/36,199 (55)	127,437/379,520 (33.2)	<.001
Asthma	262,158/488,596 (53.7)	N/A	N/A
Chronic pulmonary disease vs chronic respiratory disease	148,268/205,107 (72.3)	1,533,903/3,108,241 (48.8)	N/A
Chronic kidney disease	199,248/267,091 (74.6)	175,415/342,661 (50.6)	<.001
Liver disease vs chronic liver disease	52,271/107,984 (48.4)	212,048/562,410 (37.3)	<.001
Diabetes	284,280/407,228 (69.8)	994,675/1,607,996 (61.2)	<.001
Immunosuppression	87,748/136,583 (64.2)	188,198/423,273 (44)	<.001
Neurological disease vs chronic neurological disease	214,685/366,963 (58.5)	378,349/883,590 (42.3)	N/A
Severe mental illness	21,509/66,319 (32.4)	N/A	N/A
Learning difficulties	16,219/35,327 (45.9)	N/A	N/A
Obesity vs morbid obesity	70,142/162,397 (43.2)	167,050/532,494 (30.9)	N/A

^a^Population comparisons are not provided where we used a near-match, for example, coronary heart disease with chronic heart disease.

^b^N/A: not applicable.

In the PCSC, vaccine type and brand information were available for 71.8% (358,365/498,923; 95% CI 71.7%-72%) of influenza vaccines administered to people aged 16 to 64 years (Table 4 and Table S3 in Multimedia Appendix 1) and 81.9% (748,312/913,695; 95% CI 81.8%-82%) of those administered to people aged 65 years or older (Table 5 and Table S3 in Multimedia Appendix 1). This compares with national data where only 23.6% (696,880/2,900,000) of seasonal influenza vaccinations for people aged 16 to 64 years (Table 4) and 17.8% (1,385,888/7,700,000) for those aged 65 years or older were coded with a defined vaccine type in 2019-2020 (Table 5) [35].

**Table 4:** Type of influenza vaccine given to people aged 16-64 years who received an influenza vaccine in the Primary Care Sentinel Cohort (PCSC) and national data (as available).

Vaccine type	Vaccine recipients in PCSC 2019-2020 (n=498,923), n (%)	Vaccine recipients in national data [35] (n=2,900,000), n (%)
IIV4^a^ (unspecified)	9281 (1.9)	564,000 (19.1)
IIV4e^b^	307,617 (61.7)	N/A^c^
IIV4c^d^	41,233 (8.3)	132,000 (4.5)
IIV3^e^	234 (0)	40 (0)
Missing brand or vaccine type	140,558 (28.2)	2,200,000 (76.4)

^a^IIV4: quadrivalent inactivated influenza vaccine.

^b^IIV4e: egg-based quadrivalent inactivated influenza vaccine.

^c^N/A: Not available.

^d^IIV4c: cell-based quadrivalent inactivated influenza vaccine.

^e^IIV3: nonadjuvanted (standard) trivalent inactivated influenza vaccine.

**Table 5:** Type of influenza vaccine given to people aged 65 years or older who received an influenza vaccine in the Primary Care Sentinel Cohort (PCSC) and national data (as available).

Vaccine type	Vaccine recipients in PCSC 2019-2020 (n=913,695), n (%)	Vaccine recipients in national data [35] (n=7,700,000), n (%)
IIV4^a^ (unspecified)	2288 (0.3)	19,000 (0.2)
IIV4e^b^	4331 (0.5)	N/A^c^
IIV4c^d^	59,563 (6.5)	251,000 (3.2)
aIIV3^e^	680,961 (74.5)	1,100,000 (14.3)
IIV3^f^ (unspecified)	1169 (0.1)	3000 (0)
Missing brand or vaccine type	165,383 (18.1)	6,400,000 (82.2)

^a^IIV4: quadrivalent inactivated influenza vaccine.

^b^IIV4e: egg-based quadrivalent inactivated influenza vaccine.

^c^N/A: Not available.

^d^IIV4c: cell-based quadrivalent inactivated influenza vaccine.

^e^aIIV3: adjuvanted trivalent inactivated influenza vaccine.

^f^IIV3: nonadjuvanted (standard) trivalent inactivated influenza vaccine.

### Timing of Influenza Vaccination in PCSC

**Figure 2:**
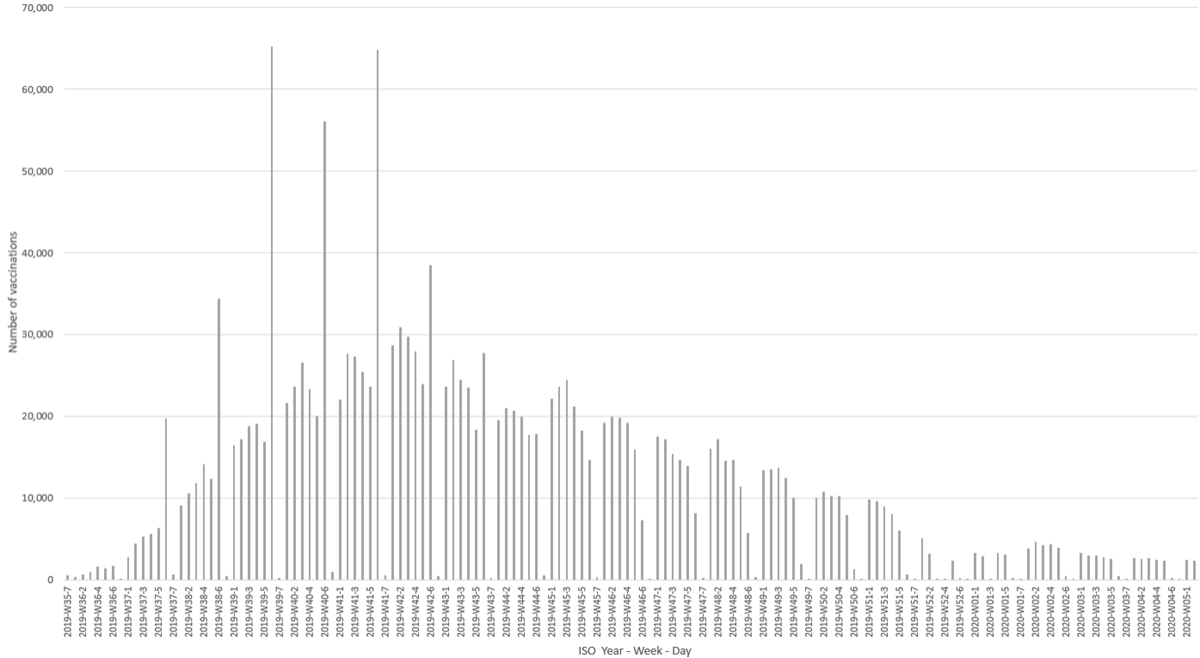
Timing of influenza vaccination across all ages in the Primary Care Sentinel Cohort. ISO: International Standard Organization.

**Figure 3:**
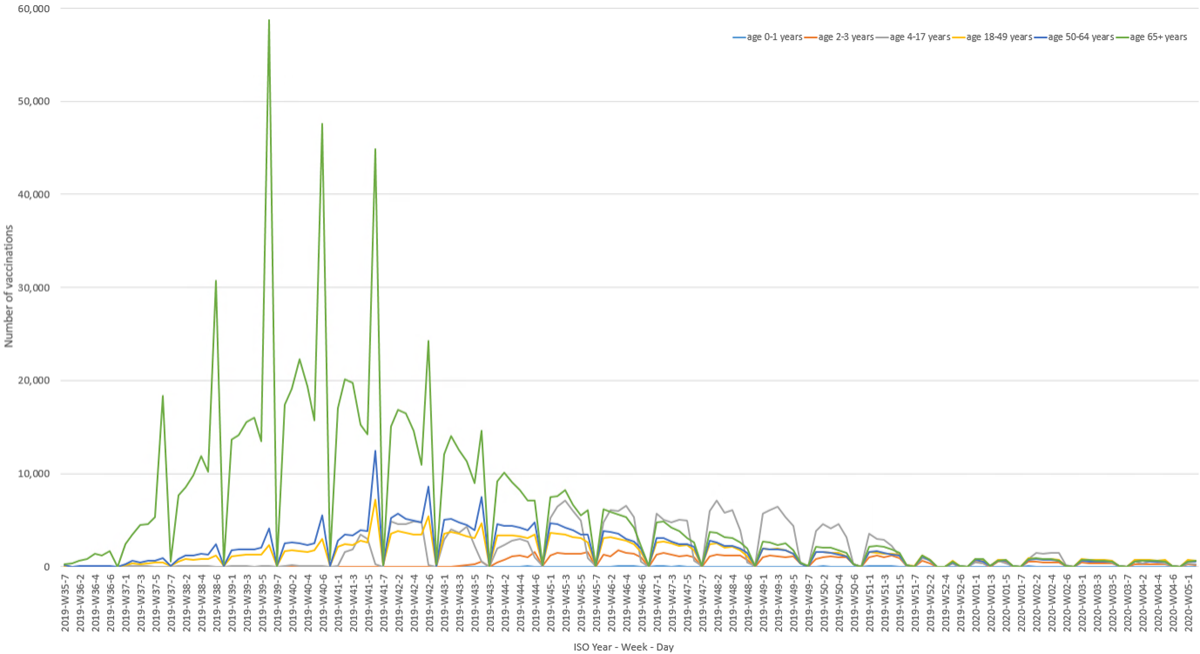
Timing of influenza vaccination by age group (in years) in the Primary Care Sentinel Cohort. ISO: International Standard Organization.

During the 2019-2020 influenza season in the PCSC, influenza vaccination started in ISO week 35 and continued until ISO week 3. Peak of vaccinations occurred in ISO week 41 (Figures 2 and 3). Weekly peaks in vaccination coverage occurred on Saturdays, especially among those aged 65 years or older. People aged 65 years or older received their influenza vaccinations earlier in the season than other age groups, with a peak around ISO week 40.

## Discussion

### Principal Findings

The population of patients in the English national PCSC is broadly comparable to the general population in terms of demographic features. However, there is a greater proportion of people aged 18-49 years in the PCSC, and people who are more deprived are underrepresented and those in the least deprived quintile overrepresented. It is possible this may result in denominator inflation in the younger half of the working-age population for any influenza vaccine coverage estimates.

Overall, influenza vaccine coverage is comparable in the PCSC to national data, although coverage of risk groups was higher in the PCSC than in comparable published national data. Influenza vaccine type was recorded for a higher proportion of vaccines recorded in the PCSC than available from national data, although there are still a substantial number of vaccinations in the PCSC for which no influenza vaccine type or brand information was recorded.

The timing of influenza vaccination during the 2019-2020 season showed that influenza vaccination started in ISO week 35, with people aged 65 years or older receiving their vaccinations earlier in the season than other age groups. Weekly peaks in vaccination coverage were seen on Saturdays, especially among those aged 65 years or older.

### Implications of the Findings

Our study demonstrated the representativeness of the PCSC in describing vaccine exposure, including timing, vaccine type, and brand-specific exposure. However, although we took steps to avoid denominator inflation, it is possible that our findings may be affected by an inflated denominator in the younger half of the working-age population (age 18 to 49 years). Denominator inflation may be a result of systematic differences in PCSC practices versus those practices outside the PCSC that have not been possible to quantify in this study, including opening times, accessibility, range of services offered, and patient satisfaction.

PCSC data about individual vaccine exposure, if combined with data on infection status, could be used to estimate vaccine effectiveness. The timing of vaccination in relation to the start of the season would allow comparison of individual vaccine exposure with circulating influenza virus strains. Where a larger population is required for a study, we could also include the RSC’s SSGPs. As the aggregated PCSC and SSGP population is over 19 million (32% of the national population; Figure 1), their combined representativeness would support a national study for which a larger sample size was needed. The RSC could also link individual exposure data to clinical records or conduct enhanced surveillance to monitor adverse events of interest.

The PCSC data did not capture the influenza vaccine type and brand for all vaccinations. Steps should be taken to improve data quality to enable comparative influenza vaccine effectiveness studies to be conducted. Of note for researchers looking to collect further information at the time of vaccination, many general practices run their influenza vaccination clinics on Saturdays, particularly for older people.

### Strengths and Limitations

Strengths of the study included its large sample size from a well-established sentinel network that includes primary care practitioners who have been recording information on influenza incidence and vaccinations for over 50 years [14]. Additionally, given the completeness of our data on influenza vaccine type, we were able to include a wider range of influenza vaccine types in the PCSC than comparable national data [35]. However, the limited amount of comparable national data also curtailed our quantitative comparison of influenza vaccine coverage. It was especially difficult to compare the proportions of those vaccinated within at-risk groups as there was no detailed information available in national data. We have presented the proportion of people in at-risk groups vaccinated against influenza as a proportion of all the people in the at-risk group who were registered that year. For example, 284,280 people with diabetes received an influenza vaccine versus 407,228 people with diabetes. Where we identified a higher proportion of risk groups, we think this likely indicated better data quality; however, this may be a statistical artifact of differences in disease prevalence. Finally, we could not precisely match some risk groups.

The COVID-19 pandemic has resulted in interruptions of health services since early 2020. The indirect effects this had on routine vaccinations in primary care may have also affected the results of this study. However, the influenza vaccination programs had largely been completed by early 2020, and we felt confident in including 2019-2020 in our analysis. That said, this limited us to including only 1 year of data in our analysis. Furthermore, a large number of doses of influenza vaccines are administered by pharmacies and other community health care workers [40]. Although the majority of these vaccines are recorded in the CMR, communications from pharmacists to GPs around vaccination records have been described as suboptimal, especially with regard to data on manufacturer or batch number [34]. This may have affected the completeness and accuracy of our data, specifically, incomplete data about influenza vaccinations provided by pharmacies and other community health care workers would serve to underestimate the vaccine coverage rate we present in this paper.

### Comparison With Prior Work

Other authors have reported strengths and limitations in the personal demographics service (described in detail in our method) and NHS number as a unique identifier [41]. Although a national identifier is overall a great strength, we need to conduct further studies to explore whether there is any denominator inflation in the PCSC database.

Past work codifying COVID-19 vaccination records in primary care settings has shown a high level of completeness of vaccine brand recording for this vaccination program. The National Immunization Management Service has recorded 99.6% of vaccine administration details and electronically transmits brand information to every individual’s GP record on a daily basis [42]. This compares with between 43.8% and 81.9% of influenza vaccine records having vaccine type and brand information accessible in the PCSC, depending on age.

Influenza vaccine effectiveness assessment is increasingly stratified by vaccine type or even vaccine brand to enable comparisons of different formulations that help to inform public health decision-making [43,44].

The United Kingdom is a possible candidate country to source comparative data for the newer influenza vaccine formulations, as it has historically had high annual influenza vaccine coverage for those aged 65 years or older. Between the 2004-2005 and 2019-2020 seasons, average coverage ranged from 71% to 75%; these figures are relatively high compared with the rest of Europe [45,46]. The World Health Organization has set a vaccination coverage target of at least 75% in the older adult population and among risk groups [47]. Influenza vaccine coverage among at-risk groups <65 years of age, excluding pregnant women, in England has been lower—approximately 49% between the 2007-2008 and 2016-2017 seasons [48,49].

### Conclusions

The English PCSC was broadly representative of the national population. It included high-quality routine data, in terms of sociodemographic risk groups, recording of risk groups, and influenza uptake compared with national data sources. However, although data quality was reported to be good, there were significant gaps in vaccine type and brand. Methods implemented to capture COVID-19 vaccine data and encode it within general practice CMRs should now be implemented for influenza vaccines. The PCSC of the RSC, and the UK health care ecosystem itself, provide favorable environments for conducting influenza vaccine benefit-risk studies.

## Appendix

Multimedia Appendix 1 Systematized Nomenclature of Medicine codes, influenza vaccine coverage by sociodemographic characteristics, and influenza vaccine exposure by type.

## Abbreviations

aIIV3: adjuvanted trivalent inactivated influenza vaccine
CMR: computerized medical record
GP: general practitioner
HD-IIV3: high-dose trivalent inactivated influenza vaccine
HD-IIV4: high-dose quadrivalent inactivated influenza vaccine
IIV: inactivated influenza vaccine
IIV4c: cell-based quadrivalent inactivated influenza vaccine
IMD: Index of Multiple Deprivation
ISO: International Standard Organization
JCVI: Joint Committee on Vaccination and Immunization
LSOA: Lower Layer Super Output Area
NHS: National Health Service
ONS: Office for National Statistics
PCSC: Primary Care Sentinel Cohort
RCGP: Royal College of General Practitioners
RSC: Research and Surveillance Centre
SSGPs: Syndromic Surveillance General Practices
UKHSA: UK Health Security Agency

## References

[1] Barberis I, Myles P, Ault SK, Bragazzi NL, Martini M (2016). History and evolution of influenza control through vaccination: from the first monovalent vaccine to universal vaccines. J Prev Med Hyg.

[2] Hannoun C (2013). The evolving history of influenza viruses and influenza vaccines. Expert Rev Vaccines.

[3] Pebody RG, Warburton F, Andrews N, Sinnathamby M, Yonova I, Reynolds A, Robertson C, Cottrell S, Sartaj M, Gunson R, Donati M, Moore C, Ellis J, de Lusignan S, McMenamin J, Zambon M (2018). Uptake and effectiveness of influenza vaccine in those aged 65 years and older in the United Kingdom, influenza seasons 2010/11 to 2016/17. Euro Surveill.

[4] Pebody RG, Whitaker H, Ellis J, Andrews N, Marques DFP, Cottrell S, Reynolds AJ, Gunson R, Thompson C, Galiano M, Lackenby A, Robertson C, O'Doherty MG, Owens K, Yonova I, Shepherd SJ, Moore C, Johnston J, Donati M, McMenamin J, de Lusignan S, Zambon M (2020). End of season influenza vaccine effectiveness in primary care in adults and children in the United Kingdom in 2018/19. Vaccine.

[5] Bruxvoort KJ, Luo Y, Ackerson B, Tanenbaum HC, Sy LS, Gandhi A, Tseng HF (2019). Comparison of vaccine effectiveness against influenza hospitalization of cell-based and egg-based influenza vaccines, 2017-2018. Vaccine.

[6] European Centre for Disease Prevention and Control (2020). Systematic Review of the Efficacy, Effectiveness and Safety of Newer and Enhanced Seasonal Influenza Vaccines for the Prevention of Laboratory-Confirmed Influenza in Individuals Aged 18 Years and Over.

[7] (2020). Joint Committee on Vaccination and Immunisation Advice on Influenza Vaccines for 2021/22.

[8] (2018). Joint Committee on Vaccination and Immunisation Advice on Influenza Vaccines for 2019/20.

[9] Powis S (2019). Update on vaccines for 2019/20 seasonal flu vaccination programme. NHS England.

[10] (2021). Advice on influenza vaccines for 2022/23. Joint Committee on Vaccination and Immunisation.

[11] de Lusignan S, Tsang RSM, Amirthalingam G, Akinyemi O, Sherlock J, Tripathy M, Deeks A, Ferreira F, Howsam G, Hobbs FDR, Joy M (2021). Adverse events of interest following influenza vaccination, a comparison of cell culture-based with egg-based alternatives: English sentinel network annual report paper 2019/20. Lancet Reg Health Eur.

[12] Bernal JL, Sinnathamby MA, Elgohari S, Zhao H, Obi C, Coughlan L, Lampos V, Simmons R, Tessier E, Campbell H, McDonald S, Ellis J, Hughes H, Smith G, Joy M, Tripathy M, Byford R, Ferreira F, de Lusignan S, Zambon M, Dabrera G, Brown K, Saliba V, Andrews N, Amirthalingam G, Mandal S, Edelstein M, Elliot AJ, Ramsay M (2021). The impact of social and physical distancing measures on COVID-19 activity in England: findings from a multi-tiered surveillance system. Euro Surveill.

[13] Correa A, Hinton W, McGovern A, van Vlymen J, Yonova I, Jones S, de Lusignan S (2016). Royal College of General Practitioners Research and Surveillance Centre (RCGP RSC) sentinel network: a cohort profile. BMJ Open.

[14] de Lusignan S, Correa A, Smith GE, Yonova I, Pebody R, Ferreira F, Elliot AJ, Fleming D (2017). RCGP research and surveillance centre: 50 years' surveillance of influenza, infections, and respiratory conditions. Br J Gen Pract.

[15] Pebody R, Warburton F, Ellis J, Andrews N, Potts A, Cottrell S, Johnston J, Reynolds A, Gunson R, Thompson C, Galiano M, Robertson C, Mullett D, Gallagher N, Sinnathamby M, Yonova I, Moore C, McMenamin J, de Lusignan S, Zambon M (2016). Effectiveness of seasonal influenza vaccine in preventing laboratory-confirmed influenza in primary care in the United Kingdom: 2015/16 mid-season results. Euro Surveill.

[16] Pebody RG, Warburton F, Ellis J, Andrews N, Thompson C, von Wissmann B, Green HK, Cottrell S, Johnston J, de Lusignan S, Moore C, Gunson R, Robertson C, McMenamin J, Zambon M (2015). Low effectiveness of seasonal influenza vaccine in preventing laboratory-confirmed influenza in primary care in the United Kingdom: 2014/15 mid-season results. Euro Surveill.

[17] de Lusignan S, Jones N, Dorward J, Byford R, Liyanage H, Briggs J, Ferreira F, Akinyemi O, Amirthalingam G, Bates C, Bernal JL, Dabrera G, Eavis A, Elliot AJ, Feher M, Krajenbrink E, Hoang U, Howsam G, Leach J, Okusi C, Nicholson B, Nieri P, Sherlock J, Smith G, Thomas M, Thomas N, Tripathy M, Victor W, Williams J, Wood I, Zambon M, Parry J, O'Hanlon S, Joy M, Butler C, Marshall M, Hobbs FDR (2020). The Oxford royal college of general practitioners clinical informatics digital hub: protocol to develop extended COVID-19 surveillance and trial platforms. JMIR Public Health Surveill.

[18] de Lusignan S, Bernal JL, Byford R, Amirthalingam G, Ferreira F, Akinyemi O, Andrews N, Campbell H, Dabrera G, Deeks A, Elliot AJ, Krajenbrink E, Liyanage H, McGagh D, Okusi C, Parimalanathan V, Ramsay M, Smith G, Tripathy M, Williams J, Victor W, Zambon M, Howsam G, Nicholson BD, Brown VT, Butler CC, Joy M, Hobbs FDR (2021). Influenza and respiratory virus surveillance, vaccine uptake, and effectiveness at a time of cocirculating COVID-19: protocol for the English primary care sentinel system for 2020-2021. JMIR Public Health Surveill.

[19] de Lusignan S, Borrow R, Tripathy M, Linley E, Zambon M, Hoschler K, Ferreira F, Andrews N, Yonova I, Hriskova M, Rafi I, Pebody R (2019). Serological surveillance of influenza in an English sentinel network: pilot study protocol. BMJ Open.

[20] Whitaker HJ, Tsang RSM, Byford R, Andrews NJ, Sherlock J, Pillai PS, Williams J, Button E, Campbell H, Sinnathamby M, Victor W, Anand S, Linley E, Hewson J, DArchangelo S, Otter AD, Ellis J, Hobbs RFD, Howsam G, Zambon M, Ramsay M, Brown KE, de Lusignan S, Amirthalingam G, Bernal JL (2022). Pfizer-BioNTech and Oxford AstraZeneca COVID-19 vaccine effectiveness and immune response amongst individuals in clinical risk groups. J Infect.

[21] Liyanage H, Akinyemi O, Pathirannahelage S, Joy M, de Lusignan S (2020). Near real time feedback of seasonal influenza vaccination and virological sampling: dashboard utilisation in a primary care sentinel network. Stud Health Technol Inform.

[22] Pathirannehelage S, Kumarapeli P, Byford R, Yonova I, Ferreira F, de Lusignan S (2018). Uptake of a dashboard designed to give realtime feedback to a sentinel network about key data required for influenza vaccine effectiveness studies. Stud Health Technol Inform.

[23] Morbey R, Smith G, Oliver I, Edeghere O, Lake I, Pebody R, Todkill D, McCarthy N, Elliot AJ (2021). Evaluating multi-purpose syndromic surveillance systems—a complex problem. Online J Public Health Inform.

[24] Tippu Z, Correa A, Liyanage H, Burleigh D, McGovern A, van Vlymen J, Jones S, de Lusignan S (2017). Ethnicity recording in primary care computerised medical record systems: an ontological approach. J Innov Health Inform.

[25] Payne RA, Abel GA (2012). UK indices of multiple deprivation—a way to make comparisons across constituent countries easier. Health Stat Q.

[26] Niggebrugge A, Haynes R, Jones A, Lovett A, Harvey I (2005). The index of multiple deprivation 2000 access domain: a useful indicator for public health?. Soc Sci Med.

[27] Regional teams. NHS England.

[28] (2019). The national flu immunisation programme 2019/20. Department of Health and Social Care.

[29] (2020). Population estimates for the UK, England and Wales, Scotland and Northern Ireland: mid-2019. Office for National Statistics.

[30] (2012). Ethnicity and national identity in England and Wales: 2011. Office for National Statistics.

[31] Detailed information on the administrative structure within England. Office for National Statistics.

[32] (2020). Quality and outcomes framework, 2019-20. NHS Digital.

[33] de Lusignan S, van Weel C (2006). The use of routinely collected computer data for research in primary care: opportunities and challenges. Fam Pract.

[34] de Lusignan S, Hoghton M, Rafi I (2017). Flu vaccination by pharmacists leads to suboptimal medical records. BMJ.

[35] (2020). Seasonal influenza vaccine uptake in GP patients: winter season 2019 to 2020; final data for 1 September 2019 to 29 February 2020. Public Health England.

[36] Wallis S (2013). Binomial confidence intervals and contingency tests: mathematical fundamentals and the evaluation of alternative methods. J Quant Linguist.

[37] Armitage P, Berry G, Matthews JNS (1994). Statistical Methods in Medical Research. 4th Edition.

[38] (2018). R FAQ. CRAN.

[39] Do i need NHS REC review?. Medical Research Council Health Research Authority.

[40] Perman S, Kwiatkowska RM, Gjini A (2018). Do community pharmacists add value to routine immunization programmes? a review of the evidence from the UK. J Public Health (Oxf).

[41] Stagg HR, Jones J, Bickler G, Abubakar I (2012). Poor uptake of primary healthcare registration among recent entrants to the UK: a retrospective cohort study. BMJ Open.

[42] Curtis HJ, Inglesby P, Morton CE, MacKenna B, Green A, Hulme W, Walker AJ, Morley J, Mehrkar A, Bacon S, Hickman G, Bates C, Croker R, Evans D, Ward T, Cockburn J, Davy S, Bhaskaran K, Schultze A, Rentsch CT, Williamson EJ, Rowan A, Fisher L, McDonald HI, Tomlinson L, Mathur R, Drysdale H, Eggo RM, Wing K, Wong AY, Forbes H, Parry J, Hester F, Harper S, O'Hanlon S, Eavis A, Jarvis R, Avramov D, Griffiths P, Fowles A, Parkes N, Douglas IJ, Evans SJ, Smeeth L, Goldacre B (2022). Trends and clinical characteristics of COVID-19 vaccine recipients: a federated analysis of 57.9 million patients' primary care records in situ using OpenSAFELY. Br J Gen Pract.

[43] Stuurman AL, Biccler J, Carmona A, Descamps A, Díez-Domingo J, Quiles CM, Nohynek H, Rizzo C, Riera-Montes M (2021). Brand-specific influenza vaccine effectiveness estimates during 2019/20 season in Europe—results from the DRIVE EU study platform. Vaccine.

[44] Stuurman AL, Ciampini S, Vannacci A, Bella A, Rizzo C, Muñoz-Quiles C, Pandolfi E, Liyanage H, Haag M, Redlberger-Fritz M, Bonaiuti R, Beutels P (2021). Factors driving choices between types and brands of influenza vaccines in general practice in Austria, Italy, Spain and the UK. PLoS One.

[45] (2021). Adult flu vaccination coverage. Nuffield Trust.

[46] de Lusignan S, Correa A, Ellis J, Pebody R (2016). Influenza vaccination: in the UK and across Europe. Br J Gen Pract.

[47] (2016). Fact sheet influenza (seasonal). World Health Organization.

[48] Ramsey M (2013). Immunisation Against Infectious Disease and Children's Health.

[49] Kohli MA, Maschio M, Mould-Quevedo JF, Ashraf M, Drummond MF, Weinstein MC (2021). The cost-effectiveness of expanding vaccination with a cell-based influenza vaccine to low risk adults aged 50 to 64 years in the United Kingdom. Vaccines (Basel).

