# Supplementary material for: A Profile of Influenza Vaccine Coverage for 2019-2020: Database Study of the English Primary Care Sentinel Cohort
Source: JMIR Public Health Surveill. 2024 May 24;10:e39297. doi: 10.2196/39297 (PMC11161707; doi:10.2196/39297)
Supplement: Multimedia Appendix 1 [file publichealth_v10i1e39297_app1.docx]

**Table S1. SNOMED codes used to identify influenza administered vaccinations**

| **SNOMED clinical term identifier** | **Description** |
| --- | --- |
| 86198006 | Administration of vaccine product containing only Influenza virus antigen (procedure) |
| 73701000119109 | Influenza vaccination given (situation) |
| 380741000000101 | First pandemic influenza vaccination given by other healthcare provider (finding) |
| 380771000000107 | Second pandemic influenza vaccination given by other healthcare provider (finding) |
|  | Influenza vaccination given by other healthcare provider (situation)SCTID: 955641000000103 |
| 955691000000108 | Seasonal influenza vaccination given by pharmacist (situation) |
| 1066171000000108 | Seasonal influenza vaccination given by midwife (situation) |

Abbreviation: SNOMED, systematized nomenclature of medicine

**Table S2. Influenza vaccine coverage in strata of age, sex, race, IMD, and region in the PCSC compared with national data in 2019-2020 (extended table)**

|  | **PCSC** | **National data [35]** |
| --- | --- | --- |
| **Age (years, inclusive)** |  |  |
| 6 months to 1 year | NA | 5,127 (5,127/932,265 = 0.6%, 95% CI, 0.5%-0.6%) |
| 0-1 | 1,216 (1,216/158,484 = 0.8%, 95% CI, 0.7%-0.8%) | NA |
| 2-3 | 63,532 (63,532/157,857 = 40.3%, 95% CI, 40.0%-40.5%) | NA |
| 2-4 | 94,290 (94,290/237,848 = 39.6%, 95% CI, 39.5%-39.8%) | 733,861 (733,861/2,013,990 = 36.4%, 95% CI, 36.4%-36.5%) |
| 4-17 | 259,902 (259,902/1,126,575 = 23.1%, 95% CI, 23.0%-23.2%) | NA |
| 5-15 | 222,938 (222,938/895,423 = 24.9%, 95% CI, 24.8-25.0%) | 1,876,012 (1,876,012/7,719,210 = 24.3%, 95% CI, 24.3%-24.3%) |
| 18-49 | 216,040 (216,040/ 2,982,390 = 7.2%, 95% CI, 7.2%-7.3%) | NA |
| 16-64 | 498,923 (498,923/4,454,172 = 11.2%, 95% CI, 11.2%-11.2%) | 4,232,160 (4,232,160/ 38,575,609 = 11.0%, 95% CI, 11.0%-11.0%) |
| 50-64 | 276,677 (276,677/1,320,621 = 21.0%, 95% CI, 20.9%-21.0%) | NA |
| ≥65 | 913,695 (913,695/1,264,700 = 72.3%, 95% CI, 72.2%-72.3%) | 7,621,505 (7,621,505/10,523,854 = 72.4%, 95% CI, 72.4%-72.5%) |
| All ages | 1,731,062 (1,731,062/7,010,627 = 24.7%, 95% CI, 24.7%-24.7%) | 14,468,665 (14,468,665/ 59,764,928 = 24.2%, 95% CI, 24.2%-24.2%) |
| **Sex** |  |  |
| Male | 790,660 (790,660/ 3,488,789 = 22.7%, 95% CI, 22.6%-22.7%) | NA |
| Female | 940,402 (940,402/ 3,521,838 = 26.7%, 95% CI, 26.7%-26.8%) | NA |
| **Race** |  |  |
| White | 1,325,892 (1,325,892/4,674,668 = 28.4%, 95% CI, 28.3%-28.4%) | NA |
| Black | 29,884 (29,884/210,501 = 14.2%, 95% CI, 14.1%-14.4%) | NA |
| Asian | 82,293 (82,293/496,330 = 16.6%, 95% CI, 16.5%-16.7%) | NA |
| Mixed | 16,797 (16,797/ 114,846 = 14.6%, 95% CI, 14.4%-14.8%) | NA |
| Missing/ other | 276,196 (276,196/1,514,282 = 18.2%, 95% CI, 18.2%-18.3%) | NA |
| **IMD quintile** |  |  |
| 1 (most deprived) | 246,536 (246,536/1,245,558 =19.8%, 95% CI, 19.7%-19.9%) | NA |
| 2 | 297,114 (297,114/1,353,953 = 21.9%, 95% CI, 21.9%-22.0%) | NA |
| 3 | 351,594 (351,594/1,409,593 = 24.9%, 95% CI, 24.9%-25.0) | NA |
| 4 | 390,403 (390,403/1,451,445 = 26.9%, 95% CI, 26.8%-27.0%) | NA |
| 5 (least deprived) | 444,876 (444,876/1,548,132 = 28.7%, 95% CI, 28.7%-28.8%) | NA |
| **NHS region [27]** |  |  |
| London | 148,930 (148,930/1,025,002 = 14.5%, 95% CI, 14.5%-14.6%) | NA |
| East of England | 131,758 (131,758/ 512,122 = 25.7%, 95% CI, 25.6%-25.9%) | NA |
| Midlands | 256,769 (256,769/1,000,656 = 25.7%, 95% CI, 25.6%-25.8%) | NA |
| North East and Yorkshire | 226,792 (226,792/823,102 = 27.6%, 95% CI, 27.5%-27.7%) | NA |
| North West | 277,648 (277,648/1,077,80 8= 25.8%, 95% CI,25.7%-25.8%) | NA |
| South East | 337,225 (337,225/1,339,701 = 25.2%, 95% CI, 25.1%-25.3%) | NA |
| South West | 351,940 (351,940/1,232,236 = 28.6%, 95% CI, 28.5%-28.6%) | NA |
| **Risk factors** |  |  |
| Coronary heart disease/ chronic heart disease | 419,417 (419,417/647,526 = 64.8%, 95% CI, 64.7%-64.9%) | 428,174 (428,174/ 1,042,670 = 41.1%, 95% CI, 41.0%-41.2%) |
| Asplenia | 19,895 (19,895/36,199 = 55.0%, 95% CI, 54.5%-55.5%) | 126,034 (126,034/ 379,520 =33.2%, 95% CI, 33.1%-33.4%) |
| Asthma | 262,158 (262,158/488,596 = 53.7% 95% CI, 53.5%-53.8%) | NA |
| Chronic pulmonary/ respiratory disease | 148,268 (148,268/205,107 = 72.3%, 95% CI, 72.1%-72.5%) | 1,517,019 (1,517,019/3,108,241=48.8%, 95% CI, 48.8%-48.9%) |
| Chronic kidney disease | 199,248 (199,248/267,091 = 74.6%, 95% CI, 74.4%-74.8%) | 173,484 (173,484/342,661=50.6%, 95% CI, 50.5%-50.8%) |
| Liver disease/ chronic liver disease | 52,271 (52,271/107,984 = 48.4%, 95% CI, 48.1%-48.7%) | 209,714 (209,714/562,410 = 37.3%, 95% CI, 37.2%-37.4%) |
| Diabetes | 284,280 (284,280/407,228 = 69.8%, 95% CI, 69.7%-70.0%) | 983,727(983,727/ 1,607,996 = 61.2%, 95% CI, 61.1%-61.3%) |
| Immuno­suppression | 87,748 (87,748/136,583 = 64.2%, 95% CI, 64.0%-64.5%) | 186,127 (186,127/423,273 = 44%, 95% CI, 43.8%-44.1%) |
| Neurological disease/ chronic neurological disease | 214,685 (214,685/366,963 = 58.5%, 95% CI, 58.3%-58.7%) | 374,185 (374,185/883,590 = 42.3%, 95% CI, 42.3%-42.5%) |
| Severe mental illness | 21,509 (21,509/66,319 = 32.4%, 95% CI, 32.1%-32.8%) | NA |
| Learning difficulties | 16,219 (16,219/35,327 = 45.9%, 95% CI, 45.4%-46.4%) | NA |
| Obesity/ morbid obesity | 70,142 (70,142/162,397 = 43.2%, 95% CI, 43.0%-43.4%) | 164,597 (164,597/532,494 = 30.9%, 95% CI, 30.8%-31.0%) |

Abbreviations: IMD, Index of Multiple Deprivation; NHS, National Health Service; ONS, Office for National Statistics; PCSC, primary care sentinel cohort.

**Table S3. Influenza vaccine exposure by type in the PCSC compared with national data in 2019-2020 (extended table)**

| **Vaccine type** | **PCSC** | **National data** |
| --- | --- | --- |
| **Age 16-64 years** |  |  |
| IIV4 | IIV4: 9,281 (9,281/498,923 = 1.9%, 95% CI, 1.8%-1.9%)  IIV4e: 307,617 (307,617/498923 = 61.7%, 95% CI, 61.5%-61.8%) | 564,000 (19.1%) |
| IIV4c/cell-based vaccine | 41,233 (41,233/498,923 = 8.3%, 95% CI, 8.2%-8.3%) | 132,000 (4.5%) |
| IIV3 | 234 (234/498,923 = 0.0% 95% CI, 0.0%-0.0%) | 40 (0.0%) |
| Missing brand/ vaccine type | 125,454 (125,454/498,923 = 25.1%: 95% CI, 25.0%-25.3%) | 2.2 million (76.4%) |
| **Age ≥65 years** |  |  |
| IIV4 | IIV4: 2,288 (2,288/913,695 = 0.3%, 95% CI, 0.2%-0.3%)  IIV4e: 4,331 (4,331/913,695 = 0.5%, 95% CI, 0.5%-0.5%) | 19,000 (0.2%) |
| IIV4c/cell-based vaccine | 59,563 (59,563/913,695 = 6.5%, 95% CI, 6.5%-6.6%) | 251,000 (3.2%) |
| aIIV3 | 680,961 (680,961/ 913,695 = 74.5%, 95% CI,74.4%-74.6%) | 1.1 million (14.3%) |
| IIV3 | 1,169 (1,169/ 913,695 = 0.1%, 95% CI, 0.1%-0.1%) | 3,000 (0.0%) |
| Missing brand/ vaccine type | 165,361 (165,361/913,695 = 18.1%, 95% CI, 18.0%-18.2%) | 6.3 million (82.2%) |

Abbreviations: aIIV3, adjuvanted trivalent inactivated influenza vaccine; IIV3, nonadjuvanted (standard) trivalent inactivated influenza vaccine; IIV4, quadrivalent inactivated influenza vaccine; IIV4c, cell-based IIV4; IIV4e, egg-based IIV4; ONS, Office for National Statistics; PCSC, primary care sentinel cohort.
